# The Prescriber’s Complete Handbook, Comprising the Principles of the Art of Prescribing, &c.

**Published:** 1852-12

**Authors:** 


### BIBLIOGRAPHICAL NOTICES.

*The Prescriber's Complete Handbook, comprising the principles of the art of Prescribing, &c.* By MM. TROUSSEAU AND REVEIL. Edited, with notes, by J. BIRKBECK NEVINS, M.D. London: Hippolyte Bailliere, 219 Regent street; and 290 Broadway, New York.

Under this somewhat pretending title we have a very fair translation of the admirable little *Traité de l'Art de Formuler* of Trousseau and Reveil. To the French physician and student the original must be invaluable, and this translation will be received with pleasure by many here and in Great Britain. The work was written with special reference to the Therapeutics of Trousseau and Pidoux, a fact that must be borne in mind in making any critical estimate of it. We have found it particularly interesting and useful as a companion to that excellent manual. As an independent treatise on the art of prescribing, it is too entirely French. It refers almost exclusively to the Paris Codex, and to French methods of prescribing and compounding, which differ essentially from our own. The American prescriber will find himself at a loss among the profusion of *collutoires*, *lavements*, *pommades*, *looches* and *papiers*, that are almost unknown to our pharmacy. While, therefore, the general principles and artistic rules of the book are of exceeding value, the special pharmaceutic details will be comparatively useless, on account of this difference. The thoughtful prescriber will, however, find here many hints not to be neglected, and perhaps the very strangeness of the formulæ may serve to lift him out of the ruts of daily unreflecting routine into which we are all too apt to fall in our prescribing.

The first part of the work gives us a natural-historical classification of the Materia-Medica, with a nosological table and an indication of the therapeutical classes attached. In translating this, Dr. Nevins retains the French names as principal, adding the English as subordinate. This we regard as an imperfection in his work. He should have substituted either the

English or Latin names as principal, and allowed the French to remain, if he chose, as subordinate. The merely English student, who runs his eye down the column of staring Roman capitals, will hardly recognize CHANVRE INDIEN as *Indian Hemp*, even if followed by that name in small italics. The second part contains the practical rules for the direction of the prescriber, and is the most valuable portion of the book. It is concise, clear and philosophical, and reveals in every line the masterly hand of Trousseau, by far the ablest living teacher of therapeutics. The sections on the Mutation, Elimination and Accumulation of Medicines are admirable. The same may be said of that on the circumstances which modify the dose, in which an immense amount of valuable information is compressed into a very small space. The chapter on the Combination of Medicines is borrowed almost entirely from Paris's Pharmacologia; and it must be admitted that the authors could not have gone to a better source, although they certainly might have made some acknowledgment of their indebtedness. Following this is a table of chemical incompatibilities, which will be found of much use. The "Short Sketch of Pharmacy," with its notice of the officinal preparations, is very French, and liable to the objection before urged. It is to be regretted that Dr. Nevins did not, by extensive notes, render it applicable to the formulæ of the British Pharmacopœias. The Magistral Formulary must incur the same criticism. The Toxicology is very concise, methodically arranged, and sufficient for all practical purposes.

The formulæ in this book are invariably given in English, as, in the original, they are in French. The current of professional opinion in France sets at present against Latin prescribing. Our authors merely say that "the prescription ought to be written either in the common language or in Latin," but they set the example of adopting the former. Hanin (*Cours de Mat. Med.*) is more explicit. He says, "Les formules doivent être écrites en langue vulgaire. Il-y-a une présomption ridicule de vouloir formuler en Latin: cet usage pouvait être bon quand la langue medicale ou pharmaceutique n'etait pas encore formée." We believe that Latin prescribing is almost unknown in France at this day. Whether or not we should follow their example, we are not, at present, prepared to determine, but we can assure our friends that there is much more to be said in favor of the change than very many suppose. Another thing we are glad to perceive is, that Messrs. Trousseau and Reveil do not inform us, as is usual, that our ℞ is an altered form of the astrological symbol of Jupiter. Dr. Nevins labors to defend the profession against the charge of using "dog-latin," by insisting that our $\overline{aa}$ stands for the Latin *alterius alteriusque*, and not for the Greek *ana*. We have always felt much more strongly the reproach of the cloven-footed bit of paganism which Paris taught us to believe in, and which, after all, is not true. It may be that foolish people did put the sign of Jupiter at the head of their prescriptions, but it is equally true that the imperative *Recipe* is necessary to any rendering of the sentence as written, (and could not, therefore, be substituted by a mere symbol,) and also that the sign ℞ is used in other connections to signify an abbreviation of any word commencing with R. In the oldest printed Missals the identical ℞ is employed to mark the responses. We have seen an old German book of Exorcisms (published by ecclesiastical authority,) in which the same type is used to designate the responses to the prayers and to head the formulæ of drugs for fumigation, &c. We certainly cannot suspect the authors of using astrological symbols as a caption to devotional exercises. If, then, the R with a crossed tail is susceptible of this innocent use, physicians might certainly be allowed to employ it without incurring the charge of retaining an absurd remnant of exploded superstition. We shall therefore continue to write it, with the firm conviction that it means properly simply *Recipe*, and nothing more.

Dr. Nevins has performed his part of the work skilfully and faithfully, for the most part. We regret that he did not exercise a more careful supervision over the original and correct its errors. There is a mistake as to the dose of the arsenical preparations, which it is surprising that the authors could make or that the translator could copy without detecting it. The *Solution febrifuge arsenicale du Dr. Boudin* is directed in the proportion of one part of arsenious acid to 1000 of water, and we are told that an ounce of it contains nearly half a grain of arsenious acid. The dose is stated as two teaspoonfuls for children, ten or fifteen for adults. On the same page Fowler's Solution is directed in the proportion of one part each of arsenious acid and carbonate of potassa to 100 of water, and we are told that one drachm contains three-fifths of a grain of arsenious acid, and one and one-fifth of a grain of arsenite of potassa. Yet in the next line we are informed that this solution "is used in the same doses and in the same way as the last." It is incomprehensible that Dr. Nevins should pass over so gross an error without noticing it. "Two tea-spoonfuls" of Fowler's Solution contain one and one-fifth gr. arsenious acid and two and two-fifths grs. arsenite of potassa, and yet this is the dose which an unsuspecting and literal prescriber by the book would give as the dose for a child! We beg that all who intend using this handbook as a guide in prescribing, will look up this erratum, and "when found, make a note of it."

